# Body Fat Percentage Is a Major Determinant of Total Bilirubin Independently of UGT1A1*28 Polymorphism in Young Obese

**DOI:** 10.1371/journal.pone.0098467

**Published:** 2014-06-05

**Authors:** Luís Belo, Henrique Nascimento, Michaela Kohlova, Elsa Bronze-da-Rocha, João Fernandes, Elísio Costa, Cristina Catarino, Luísa Aires, Helena Ferreira Mansilha, Petronila Rocha-Pereira, Alexandre Quintanilha, Carla Rêgo, Alice Santos-Silva

**Affiliations:** 1 Departamento de Ciências Biológicas, Faculdade de Farmácia, Universidade do Porto, Porto, Portugal; 2 Instituto de Biologia Molecular e Celular (IBMC), Universidade do Porto, Porto, Portugal; 3 Institute for Biomedical Imaging and Life Science (IBILI), Faculdade de Medicina, Universidade de Coimbra, Coimbra, Portugal; 4 Centro de Investigação em Actividade Física, Saúde e Lazer (CIAFEL), Faculdade de Desporto, Universidade do Porto, Porto, Portugal; 5 Instituto Universitário da Maia (ISMAI), Maia, Portugal; 6 Departamento da Infância e Adolescência/Serviço de Pediatria do Centro Hospitalar do Porto, Porto, Portugal; 7 Centro de Investigação em Ciências da Saúde, Universidade da Beira Interior, Covilhã, Portugal; 8 Instituto de Ciências Biomédicas Abel Salazar (ICBAS), Universidade do Porto, Porto, Portugal; 9 Centro da Criança e do Adolescente. Hospital CUF Porto, Center for Health Technology and Services Research (CINTESIS), Faculdade de Medicina, Universidade do Porto, Porto, Portugal; Tor Vergata University of Rome, Italy

## Abstract

**Objectives:**

Bilirubin has potential antioxidant and anti-inflammatory properties. The UGT1A1*28 polymorphism (TA repeats in the promoter region) is a major determinant of bilirubin levels and recent evidence suggests that raised adiposity may also be a contributing factor. We aimed to study the interaction between *UGT1A1* polymorphism, hematological and anthropometric variables with total bilirubin levels in young individuals.

**Methods:**

350 obese (mean age of 11.6 years; 52% females) and 79 controls (mean age of 10.5 years; 59% females) were included. Total bilirubin and C-reactive protein (CRP) plasma levels, hemogram, anthropometric data and *UGT1A1* polymorphism were determined. In a subgroup of 74 obese and 40 controls body composition was analyzed by dual-energy X-ray absorptiometry.

**Results:**

The *UGT1A1* genotype frequencies were 49.9%, 42.7% and 7.5% for 6/6, 6/7 and 7/7 genotypes, respectively. Patients with 7/7 genotype presented the highest total bilirubin levels, followed by 6/7 and 6/6 genotypes. Compared to controls, obese patients presented higher erythrocyte count, hematocrit, hemoglobin and CRP levels, but no differences in bilirubin or in *UGT1A1* genotype distribution. Body fat percentage was inversely correlated with bilirubin in obese patients but not in controls. This inverse association was observed either in 6/7 or 6/6 genotype obese patients. *UGT1A1* polymorphism and body fat percentage were the main factors affecting bilirubin levels within obese patients (linear regression analysis).

**Conclusion:**

In obese children and adolescents, body fat composition and *UGT1A1* polymorphism are independent determinants of total bilirubin levels. Obese individuals with 6/6 *UGT1A1* genotype and higher body fat mass may benefit from a closer clinical follow-up.

## Introduction

Bilirubin is the ultimate product of the haem group catabolism and serves as a diagnostic marker of liver and blood disorders [1]. Bilirubin is a water-insoluble compound that circulates bounded to albumin and requires glucuronidation by a microsomal enzyme, the uridine diphosphate glucuronosyltransferase (UGT) 1A1, to be excreted. The *UGT1A1* gene locus has been mapped to chromosome 2q37 [2] and one of the most common genetic variants that affects the glucuronidation of bilirubin is a TA duplication polymorphism in the TATA box region of the gene promoter. Homozygous individuals carrying the A(TA)7TAA allele have higher levels of unconjugated bilirubin (UCB), caused by a reduction of 30% in the *UGT1A1* transcription [3]. The estimated frequency of this allele is 0.35 in Caucasians, leading to a homozygous genotype in about 10% of the population, but the frequency is highly variable in different ethnicities [4], [5]. Homozygosis for the TA duplication was considered as the main cause of Gilbert syndrome in Caucasian population [3], [4], and justify some of the inter-individual variations in bilirubin levels [6].

Under certain conditions bilirubin can be toxic [7]. High plasma concentrations are associated with deleterious effects in new-borns, increasing the risk of neurological dysfunction [7], [8], as a result of its toxic effect on neuronal tissue. However, recent investigation has recognized that UCB exerts anti-oxidant and anti-inflammatory activities, and that mild hyperbilirubinaemia might have positive health effects. UCB inhibits lipid peroxidation [9] and suppresses inflammation in activated neonatal neutrophils[10], and population studies documented that individuals with higher circulating UCB have a reduced incidence of cardiovascular problems [11]–[13] and of carcinoma in general [14]. Furthermore, subjects with Gilbert syndrome seem to present low levels of oxidative stress associated with hyperbilirubinemia [15].

Obesity, a low-grade inflammatory disease [16], is increasing all over the world and is a significant risk factor for cardiovascular diseases (CVD). This is of particular concern in our country, considering the very high prevalence of overweight/obesity (31.5%) in Portuguese children when compared to other European countries [17]. In obesity, cardiovascular morbidity and mortality are associated with classic risk factors, namely dyslipidemia, hypertension and impaired glucose metabolism. These risk factors, known as predictive of CVD, are characteristic of the metabolic syndrome (MS) [18]. Moreover, serum bilirubin levels are inversely associated with the MS and systemic inflammation in adults [19]–[21], as well as in children and adolescents [22]. In particular, abdominal obesity *per se* seems to be associated with low serum bilirubin levels [21]–[23]. Furthermore, a recent study hypothesized that circulating bilirubin levels might be already altered in overweight asymptomatic middle-aged individuals before full development of the MS [24].

The aim of our work was to evaluate how total bilirubin (TB) levels are influenced by UGT1A1*28 polymorphism, haematological, biochemical and anthropometric variables in Portuguese obese children and adolescents.

## Materials and Methods

### 2.1. Subjects

Obese children and adolescents, aged 4–18 years, were identified from medical records, at the outpatient clinics of pediatric obesity in two hospitals in Porto - Portugal. A group of children from 5 primary and 2 middle and high public schools from Oporto suburban setting, were also recruited to this study, providing a control group and enlarging the obese group.

The study protocol was approved by the Committee on Ethics of Oporto Hospital Centre, the Committee on Ethics of Hospital São João, the Review Committee of the Scientific Board of the Faculty of Sport of the University of Porto as well as by the Foundation of Science and Technology.

As referred, the main objective of this study was to investigate total bilirubin levels in obese and non-obese subjects; thus, the sample size was based on this main variable. Considering the difficulty of getting blood samples from non-obese, healthy subjects, the sample size relation between obese and controls was set up as 4∶1. Assuming that a clinical relevant difference between experimental and control was 1 unit of bilirubin, and a common standard deviation of 3 units of bilirubin, for a relation of 4∶1, the sample size calculations, for a power of 80% and a significant level of 5%, define, respectively, 335 obese to 89 controls. In accordance, we tried to achieve these numbers as close as possible; a total of 350 obese children and adolescents and 79 controls participated in the study after informed and written consent of their parents. Smokers, subjects with diabetes mellitus, endocrine disorders, hereditary diseases, inflammatory or infectious diseases or under any therapy that could interfere with our results were excluded from the study.

### 2.2. Procedures and Assays

#### 2.2.1. Anthropometric characterization and clinical evaluation

All participants were subjected to clinical examination. Height and weight were measured. Obesity was defined as body mass index (BMI) z-score greater than +1.65 for age and gender, according to 2000 Centre for Disease Control and Prevention (CDC) growth charts. Body composition was evaluated by dual-energy X-ray absorptiometry (DEXA) in a subgroup of participants (74 obese and 40 controls).

#### 2.2.2. Blood samples

Blood was collected by venipuncture in EDTA containing tubes, after overnight fasting (10–12 h) and processed within 2 h of collection. Aliquots of buffy-coat and plasma were made, and immediately stored at −80°C until assayed.

#### 2.2.3. Haematological data

Red blood cell (RBC) count, haematocrit (Ht), haemoglobin (Hb) concentration and haematimetric indices [mean cell volume (MCV), mean cell Hb (MCH) and mean cell Hb concentration (MCHC)] were measured by using an automatic blood cell counter (ABX Micros 60-OT).

#### 2.2.4. DNA analysis

Genomic DNA was extracted from buffy-coat by proteinase K/salt precipitation method [25], [26]. Genotyping TA duplication in the TATA box of the UGT1A1 promoter was performed by polymerase chain reaction (PCR) (forward: 5′-TAACTTGGTGTATCGATTGGTTTTTG-3′; reverse: 5′-ACAGCCATGGCGCCTTTGCT-3′). PCR was followed by electrophoresis in 15% polyacrylamide gel in a Tris/Borate/EDTA buffer. The gel was stained with silver nitrate, photographed and samples were classified.

#### 2.2.5. Plasma analysis

The plasma levels of C-reactive protein (CRP) were determined by immunoturbidimetry [CRP (latex) High-Sensitivity, Roche Diagnostics] and quantification of TB was performed by a colorimetric test (diazotized sulfanilic acid reaction, Roche Diagnostics).

The determination of circulating levels of glucose and insulin was performed by using routine automated technology (ABX Diagnostics). Homeostasis model assessment of insulin resistance (HOMA_IR_) was calculated [27].

### 2.3. Statistical Analysis

The distributions of continuous variables were analysed using Kolmogorov-Smirnov tests. Normally distributed variables are presented as mean ± SD and those non-normally distributed are presented as median (interquartile range). Comparisons between two groups were performed using Student’s unpaired *t*-test or Mann-Whitney *U* test. Adjustment for confounding factors was performed using ANCOVA. The association between categorical variables was analysed using chi-squared (χ^2^) test and Fisher’s exact test.

The strength of the association between the variables was estimated by Pearson correlation coefficient, after log transformation of the variables (whenever necessary). To evaluate the contribution of the different variables to TB levels, multiple regression analysis was performed, using stepwise selection, with an entry criteria of *p*<0.05.

Statistical analysis was performed using the Statistical Package for Social Sciences (SPSS), version 20.0 (IBM, Armonk, NY, USA). Statistical significance was accepted at *p* less than 0.05.

## Results

The anthropometric data, *UGT1A1* genotypes and haematological parameters of the obese children and adolescents (*n* = 350) and controls (*n* = 79), according to gender, are presented in Table 1.

**Table 1:** Anthropometric data, UGT1A1*28 polymorphism, haematological and biochemical parameters of the participants in the study.

	Controls (*n* = 79)			Obese patients (*n* = 350)				
	Females	Males	*p*	Females	Males	*p*	*p* †	*p* ††
Number ofparticipants	47	32		182	168			
Age (years)	10.5±4.0	10.7±3.6	0.830	11.6±2.9	11.7±2.9	0.559	0.083	0.113
Height (cm)	139.7±17.9	143.8±17.6	0.317	151.3±13.2	155.4±15.4	0.008	<0.001	<0.001
Weight (kg)	37.0±14.6	39.7±15.8	0.440	72.1±22.5	76.2±27.4	0.128	<0.001	<0.001
BMI (kg/m^2^)	18.1±2.9	18.3±2.9	0.691	30.7±5.8	30.5±6.4	0.762	<0.001	<0.001
BMI z-score	0.17±0.65	0.24±0.77	0.636	2.22±0.34	2.30±0.40	0.046	<0.001	<0.001
Body fat (%)	30.8^a^±4.1	25.4^b^±5.2	0.001	43.5^c^±4.1	39.8^d^±6.6	0.156	<0.001	<0.001
Trunk fat (%)	25.6^a^±4.8	21.9^b^±6.0	0.045	41.1^c^±8.9	37.8^d^±7.9	0.107	<0.001	<0.001
*UGT1A1* genotype								
6/6, *n* (%)	21 (44.7%)	12 (37.5%)	0.298	92 (50.6%)	89 (53.0%)	0.433	0.455	0.085
6/7, *n* (%)	21 (44.7%)	19 (59.4%)		79 (43.4%)	64 (38.1%)			
7/7, *n* (%)	5 (10.6%)	1 (3.1%)		11 (6.0%)	15 (8.9%)			
RBC (×10^12^/L)	4.62±0.29	4.77±0.29	0.031	4.78±0.32	5.03±0.39	<0.001	0.003	<0.001
Hb (g/dL)	13.1±0.9	13.6±1.2	0.029	13.6±0.8	14.2±1.2	<0.001	0.001	0.017
Ht (L/L)	0.39±0.03	0.40±0.04	0.263	0.40±0.02	0.42±0.03	<0.001	0.033	0.003
MCV (fL)	84.9±4.6	84.0±6.1	0.486	84.2±5.1	83.8±4.7	0.454	0.432	0.846
MCH (pg)	28.4±1.7	28.6±2.0	0.684	28.5±1.7	28.2±1.6	0.185	0.909	0.239
MCHC (g/dL)	33.4±1.2	34.0±1.1	0.025	33.8±1.0	33.7±1.1	0.271	0.027	0.081
Total bilirubin (µmol/l)	8.89 (5.47–13.34)	7.52 (5.30–11.54)	0.463	8.89 (6.16–11.63)	9.23 (6.84–12.65)	0.232	0.919	0.079
Acute phase protein								
CRP (mg/L)	0.26 (0.20–0.73)	0.36 (0.26–0.83)	0.121	1.83 (0.85–3.73)	1.64 (0.85–3.54)	0.527	<0.001	<0.001
Glucose metabolism								
Glucose (mg/dl)	85.3±9.3	87.0±6.5	0.365	84.0±8.9	85.8±12.6	0.121	0.384	0.419
Insulin (µU/ml)	6.8 (5.0–9.9)	5.3 (4.1–8.5)	0.051	16.6 (11.7–23.2)	12.8 (9.1–20.0)	0.001	<0.001	<0.001
HOMA_IR_	1.41 (1.06–2.05)	1.14 (0.82–1.83)	0.130	3.39 (2.21–4.87)	2.75 (1.88–4.06)	0.006	<0.001	<0.001

Values are given as mean ± SD or median (interquartile range), unless otherwise indicated.

BMI, body mass index; RBC, red blood cells; Hb, haemoglobin; Ht, haematocrit; MCV, mean cell volume; MCH, mean cell haemoglobin; MCHC, mean cell haemoglobin concentration; CRP, C-reactive protein; HOMA_IR_, homeostasis model assessment insulin resistance.

a *n* = 25;

b *n* = 15;

c *n* = 34;

d *n* = 40.

† Controls *versus* obese patients (females).

†† Controls *versus* obese patients (males).

Comparing males and females within the control group, body fat and trunk fat percentages were significantly lower for boys, whereas RBC count, Hb levels and MCHC values were significantly higher. Within obese patients, RBC count, Hb levels and Ht values were significantly higher for boys, whereas insulin levels and HOMA_IR_ values were lower. No statistical significant differences were found in the distribution of subjects with respect to *UGT1A1* genotypes or in TB levels between boys and girls, within both groups.

Compared to controls, and as expected considering the inclusion criteria, obese patients presented significantly higher height, weight, BMI, BMI z-score, body fat and trunk fat percentages (*p*<0.001 for all). Obese patients also presented significantly higher erythrocyte count, Ht and HOMA_IR_ values and Hb, insulin and CRP levels (*p*<0.001 for all), but no significant differences in TB levels (*p* = 0.222), MCV (*p* = 0.432), MCH (*p* = 0.474), MCHC (*p* = 0.603), glucose (*p* = 0.389) or in *UGT1A1* genotype distribution (*p* = 0.244). These results were similar when groups were analysed separately according to gender (Table 1), with exception for MCHC values that were slightly (but significantly) higher in female obese patients. The “7” allele was more prevalent in male controls than in male obese patients, but this difference was not statistically significant (*p* = 0.085).

**Figure 1:**
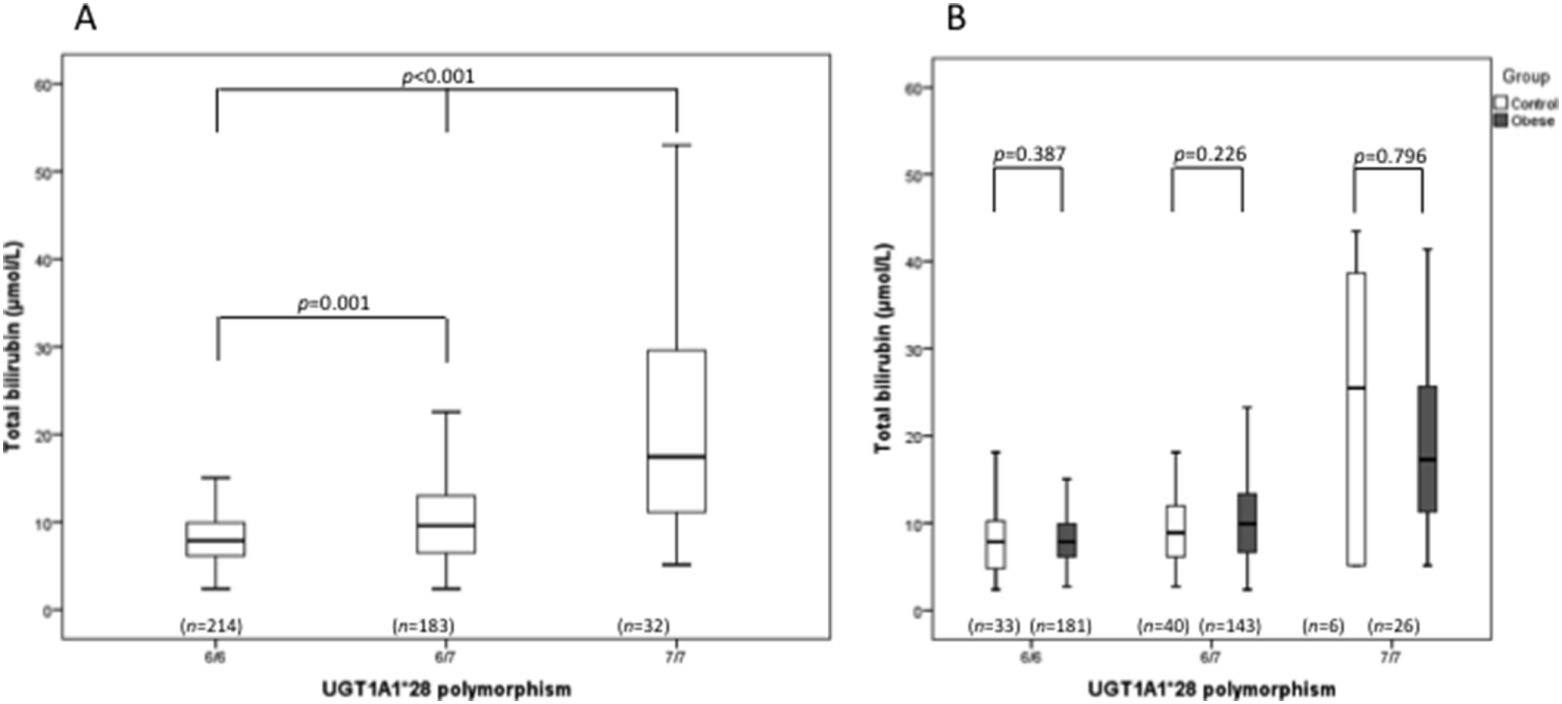
Total bilirubin levels in all participants according to the number of TA repeats in the promoter region of *UGT1A1* gene (A) and also according to group (B), control and obese. The boxes represent the interquartile range (IQR), with the upper and lower edges of the boxes representing the 75th and 25th percentiles, respectively. The central horizontal lines within the boxes represent median levels for each group. The vertical whiskers above and below the boxes represent the range of outlying data points up to 1.5 times the IQR.

The *UGT1A1* genotype frequencies in all studied individuals were 49.9%, 42.7% and 7.5% for 6/6, 6/7 and 7/7 genotypes, respectively. UGT1A1*28 polymorphism was associated with different TB levels (Figure 1A); patients with 7/7 genotype presented the highest TB levels, followed by 6/7 and 6/6 genotypes (*p*<0.01 between all groups). No significant differences in TB levels were observed between obese and control individuals, for the different *UGT1A1* genotypes (Figure 1B).

Within the control group (*n* = 79), TB levels correlated positively and significantly with age (*r* = 0.304, *p* = 0.007), height (*r* = 0.360, *p* = 0.001), weight (*r* = 0.390, *p*<0.001), BMI (*r* = 0.370, *p* = 0.001), Ht (*r* = 0.247, *p* = 0.028), MCV (*r* = 0.292, *p* = 0.009), and correlated negatively and significantly with MCHC (*r* = –0.258, *p* = 0.022). Within the obese group (*n* = 350), TB levels correlated positively and significantly with age (*r* = 0.284, *p*<0.001), height (*r* = 0.285, *p*<0.001), weight (*r* = 0.219, *p*<0.001), BMI (*r* = 0.123, *p* = 0.021), Hb (*r* = 0.305, *p*<0.001), Ht (*r* = 0.352, *p*<0.001), MCV (*r* = 0.394, *p*<0.001), MCH (*r* = 0.301, *p*<0.001) and correlated negatively and significantly with BMI z-score (*r* = –0.131, *p* = 0.014), MCHC (*r* = –0.149, *p* = 0.006) and CRP (*r* = –0.178, *p* = 0.001).

**Figure 2:**
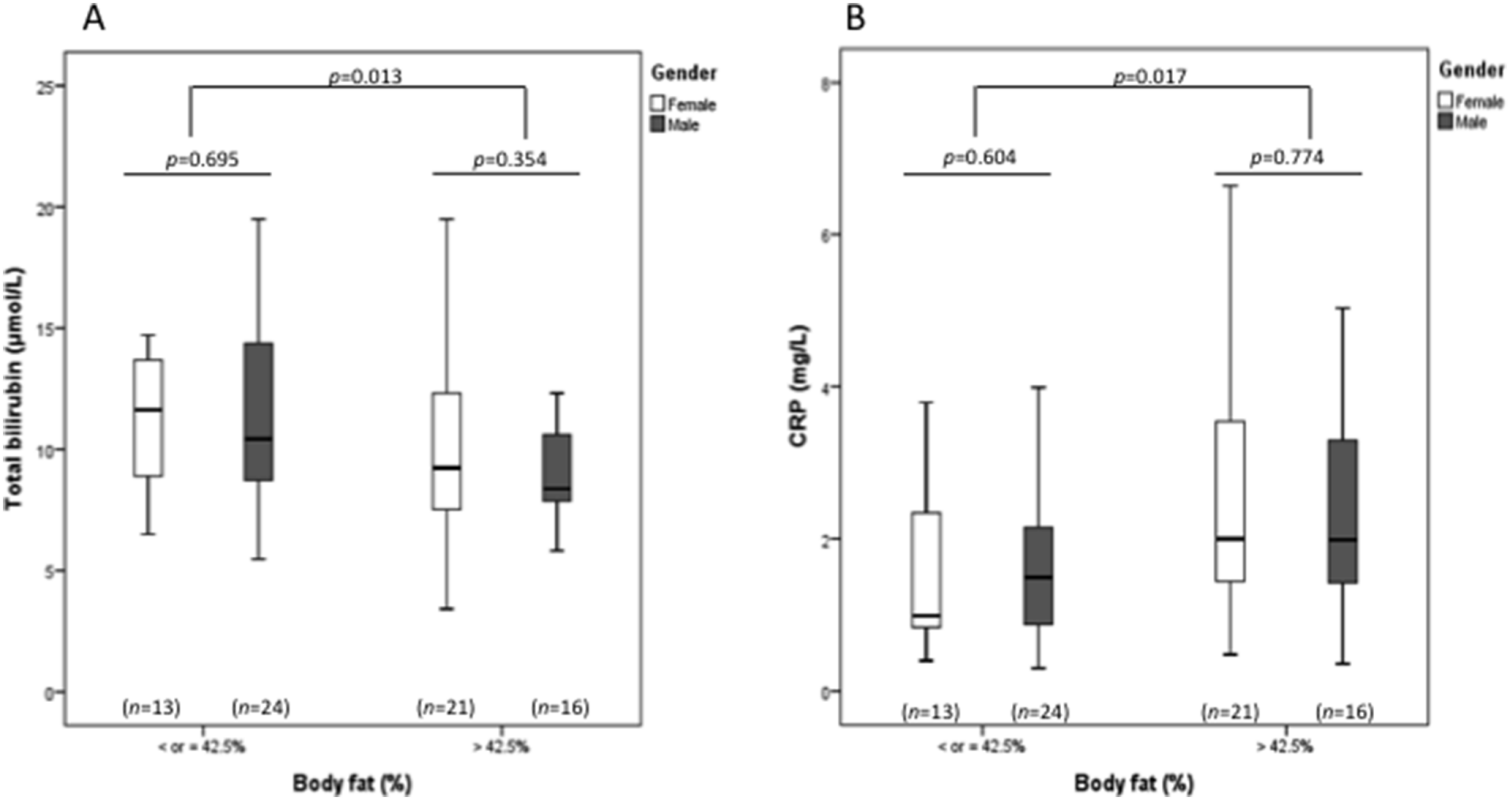
Total bilirubin (A) and C-reactive protein (B) levels in obese participants according to gender and to body fat percentage (*n* = 74), using the cut-off value of 42.5% (cut-off that corresponds to the median value for the obese group). The boxes represent the interquartile range (IQR), with the upper and lower edges of the boxes representing the 75th and 25th percentiles, respectively. The central horizontal lines within the boxes represent median levels for each group. The vertical whiskers above and below the boxes represent the range of outlying data points up to 1.5 times the IQR.

The characteristics of obese patients whose body composition was evaluated by DEXA (*n* = 74) are presented in Table 2. These obese patients were divided in two groups according on having a body fat lower or higher/equal than 42.5% (cut-off that corresponds to the median value for the obese group). The two groups of obese patients were matched for gender and *UGT1A1* genotype distribution, but not for age. Patients presenting higher body fat had lower bilirubin and higher CRP levels (Table 2). These differences were similar to both sexes (Figure 2) and remained statistically significant after adjustment for age. No significant differences in HOMA_IR_ values were found between the two groups.

**Table 2:** Anthropometric data, UGT1A1*28 polymorphism, haematological and biochemical parameters of obese patients according to body fat percentage (*n* = 74) lower or higher/equal than 42.5% (median value for the obese group).

	Obese patients (*n* = 74)		
	Body fat ≤42.5%	Body fat >42.5%	*p*
Number of participants	37	37	
Female, *n* (%)	13 (35.1%)	21 (56.8%)	0.102
Age (years)	11.0±3.0	9.5±2.5	0.022
Height (cm)	149.2±14.3	144.0±14.2	0.126
Weight (kg)	59.8±18.9	61.4±23.9	0.749
BMI (kg/m^2^)	26.0±4.0	28.4±5.5	0.041
BMI z-score	1.98±0.24	2.31±0.26	<0.001
Body fat (%)	36.8±4.3	46.1±2.6	<0.001
Trunk fat (%)	34.5±5.5	45.3±3.4	<0.001
*UGT1A1* genotype			
6/6, *n* (%)	21 (56.8%)	19 (51.4%)	0.359
6/7, *n* (%)	14 (37.8%)	18 (48.6%)	
7/7, *n* (%)	2 (5.4%)	0 (0%)	
RBC (×10^12^/L)	4.83±0.38	4.91±0.34	0.389
Hb (g/dL)	13.9±1.0	13.6±0.8	0.268
Ht (L/L)	0.41±0.03	0.41±0.02	0.798
MCV (fL)	85.4±4.9	83.8±4.6	0.156
MCH (pg)	28.7±1.7	27.8±1.5	0.017
MCHC (g/dL)	33.6±0.8	33.2±0.9	0.020
Total bilirubin (µmol/l)	11.29 (8.72–14.36)	8.89 (7.69–11.63)	0.013
Acute phase protein			
CRP (mg/L)	1.31 (0.84–2.30)	2.00 (1.43–3.54)	0.017
Glucose metabolism			
Glucose (mg/dl)	83.5±7.6	81.0±8.6	0.191
Insulin (µU/ml)	11.6 (8.9–14.6)	15.3 (7.5–22.9)	0.272
HOMA_IR_	2.25 (1.91–3.01)	3.15 (1.57–4.56)	0.361

Values are given as mean ± SD or median (interquartile range), unless otherwise indicated.

BMI, body mass index; RBC, red blood cells; Hb, haemoglobin; Ht, haematocrit; MCV, mean cell volume; MCH, mean cell haemoglobin; MCHC, mean cell haemoglobin concentration; CRP, C-reactive protein; HOMA_IR_, homeostasis model assessment insulin resistance.

Associations between body and trunk fat were only accessed in participants that evaluated their body composition by DEXA (74 obese and 40 controls). Body fat and trunk fat percentages were negatively and significantly related with TB levels in obese patients (*r* = –0.287, *p* = 0.013 and *r* = –0.245, *p* = 0.038) but not within controls (*r* = 0.012, *p* = 0.941 and *r* = 0.014, *p* = 0.002).

**Figure 3:**
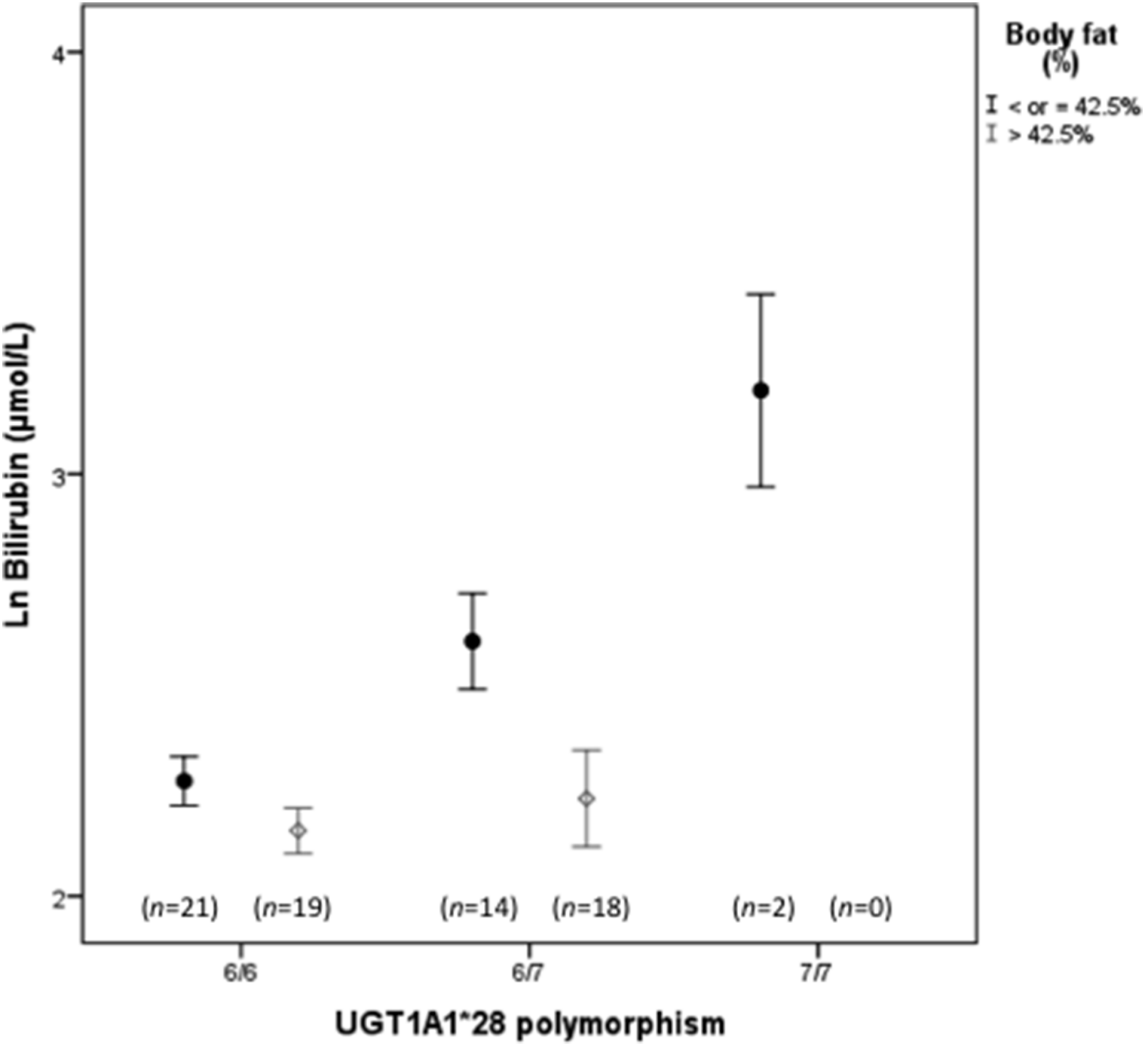
Effect of body fat percentage on total bilirubin levels according to UGT1A1*28 polymorphism on obese patients. For a better visualization of the results we used for body fat percentage a cut-off of 42.5% (cut-off that corresponds to the median value for the obese group). Results are presented as mean ± standard error of mean. The influence of body fat percentage, adjusted for *UGT1A1* polymorphism, on total bilirubin levels, was highly significant (*p* = 0.009), by multiple regression analysis.

By linear regression analysis, the UGT1A1*28 polymorphism and body weight were the only factors associated to bilirubin levels within controls (Ln TB = 1.143+0.462 UGT1A1*28 polymorphism +0.014 weight; standardised Beta: 0.598 and 0.490; *p*<0.001 and *p* = 0.001, respectively). Within obese patients, the *UGT1A1* polymorphism and body fat percentage were the main determinant factors of bilirubin levels (Ln TB = 2.761+0.251 UGT1A1*28 polymorphism −0.020 body fat; standardised Beta: 0.348, −0.291; *p* = 0.002 and *p* = 0.009, respectively). For a better visualization of the results (graphically), obese participants were divided on the basis of their *UGT1A1* genotype and on having a body fat lower or higher/equal than 42.5% (cut-off that corresponds to the median value for the obese group; Figure 3).

## Discussion

As far as we know, this is the first report assessing the concomitant influence of UGT1A1*28 polymorphism and adiposity markers on bilirubin levels in obese children and adolescents. We demonstrated that body fat percentage is a major determinant of TB levels independently of UGT1A1*28 polymorphism in obese children and adolescents.

It is known that *UGT1A1* polymorphisms are associated with bilirubin levels and our data are in agreement with previous reports in young patients and adults [15], [28]–[30]. Patients and controls with 7/7 genotype presented the highest TB levels, followed by 6/7 and 6/6 genotypes (Figure 1A and 1B).

The frequency of 7/7 homozygotes (7.5% in the whole population) was lower than that observed in other works, namely in healthy Greek [29] and Slovenian [30] populations, with frequencies of 14.8% and 13.6%, respectively. However, the distribution of subjects with respect to *UGT1A1* genotypes was similar to that found in previous studies involving Portuguese children with Hereditary Spherocytosis, with a 7/7 frequency of 8.8%(28), as well as Portuguese healthy subjects, with frequencies observed in two studies of 6.3 and 9.9% [28], [31]. Thus, it seems reasonable to assume that the frequency of 7/7 homozygotes in the Portuguese population may be lower than that observed in other Caucasian populations.

Other potential variables could influence TB levels. Within both controls and obese patients TB levels were positively and significantly correlated with age, height, weight, BMI, and Ht. However, BMI z-score, body fat and trunk fat percentages were negatively and significantly related with TB levels in obese patients, but not within controls. In multiple regression analysis, the UGT1A1*28 polymorphism and body weight were the only factors associated to bilirubin levels within controls, whereas the UGT1A1*28 polymorphism and body fat percentage were the main determinant factors of bilirubin levels within obese patients.

In the present study, the evaluation of body composition by DEXA was performed in a subgroup of participants. Despite the lower number of participants in this sub-analysis, the negative relation between bilirubin and body fat percentage was highly statistically significant and independent of the effect of UGT1A1*28 polymorphism. Furthermore, this negative relation is in agreement with a previous study involving 41 lean and obese adult men and women [23].

Bilirubin derives mainly from the haem present in Hb, released during breakdown of senescent erythrocytes [1]. Thus, in healthy conditions, it would be assumed that increases in Hb levels are generally associated with increases in TB. This explains our positive association between the age of the participants and TB, as in young individuals there is a physiological increase in Hb levels with age. It is well known that Hb and Ht increase substantially during childhood, whereas RBC count remains almost constant [32]. Differences according to gender become prominent in the second decade of life; with menstruation, these three variables tend to be lower in females. The inclusion in our study of subjects with a range of age between 4 and 18 years old justifies the higher values of RBC and Hb observed in males within both controls and obese patients (Table 1). The differences were particularly evident in obese patients, not only because of the large number of subjects included in this group but also due to the enhanced puberty development in obesity. Actually, the increasing prevalence of obesity in children worldwide is a major cause of precocious pubertal maturation, verified during the past decades [33].

Total and direct bilirubin levels were reported to be higher in normal weight adult males than in females [24], [34], [35], but similar within overweight patients [24]. In our study, we observed no statistical significant differences between boys and girls regarding TB levels, either in controls or in obese patients. Within these two groups, males and females were matched for age, weight, BMI and distribution of *UGT1A1* genotypes (Table 1) and, therefore, the analysis of TB was not greatly affected by these factors.

In the present manuscript, obese patients and controls were matched for age and distribution of *UGT1A1* genotypes, allowing the comparison of groups. Independently of gender, obese patients presented higher RBC count, Hb levels and Ht values compared with controls (Table 1). The higher weight and BMI in obese patients trigger the stimulation of erythropoiesis in order to supply adequate oxygenation to increased body tissues. The lower values of MCHC in those with higher body fat (Table 2) show a reduced hemoglobinization of the erythrocytes, suggesting an underlying disturbance in iron metabolism that might be due to a higher degree of inflammation presented by these individuals (significantly higher CRP), which may interfere with iron absorption and iron mobilization for erythropoiesis [36].

Despite higher RBC count and Hb levels in obese patients, TB levels were similar between groups. A speculative explanation to this observation is bilirubin consumption occurring in obesity, a hypothesis shared by others (24); obesity is associated with increased inflammation [16], [37], [38] and oxidative stress [39], [40], and bilirubin, presenting antioxidant and anti-inflammatory properties [9], [10], may be somewhat consumed. In fact, oxidative stress increases with increasing BMI and age [34]. In line with this, we found that bilirubin levels are negatively correlated with body and trunk fat percentages and CRP levels within obese patients. Moreover, when obese patients were divided in two groups according to the median value of body fat presented by this group (42.5%), patients presenting higher body fat presented lower bilirubin and higher CRP levels (Table 2). The negative relation that we found between bilirubin and CRP levels is in line with the bilirubin’s anti-inflammatory activity, as previously reported [41]–[44].

In obese patients, insulin resistance may also underlie the association between lower bilirubin levels and higher body fat percentages. Indeed, it seems that the activity of heme oxygenase-1, the rate-limiting enzyme of bilirubin production, is impaired in insulin resistant states [45], [46]. Also, the up-regulation of heme oxygenase-1 in adipocyte by insulin was recently demonstrated [47]. In this work obese patients presented higher HOMA_IR_ values compared to controls (Table 1). Obese patients with body fat percentages higher than 42.5% also presented higher HOMA_IR_ values, although without statistical significance. However, no significant correlation was obtained between HOMA_IR_ and bilirubin. Thus, association between insulin resistance and bilirubin might not be so clear in paediatric populations.

A previous work from our group demonstrated that BMI z-score is significantly and independently related to the lipid profile in obese children and adolescent [48]. However, in the present study BMI z-score was poorly related with TB levels in obese patients and it was not an independent predictor of bilirubin plasma concentration. This suggests that body fat percentage is a better indirect marker of oxidative stress, rather than BMI z-score. Actually BMI z-score is calculated using the BMI of patients, adjusted to age and gender, but it may not necessarily express the degree of obesity.

Individuals with a higher physical fitness index (which serves as an aerobic assessment) seem to present with higher bilirubin levels [24] and a study performed in overweight and obese adult patients demonstrated an increase in bilirubin levels due to short-term weight loss [35]. It seems that high doses of exercise training are necessary to significantly increase bilirubin levels in overweight and obese women [49]. The fact that bilirubin levels increase as a function of weight loss may be of particular importance in obese individuals with *UGT1A1* genotypes associated to lower bilirubin levels, as we here demonstrated effects on TB by body fat composition in addition to the UGT1A1*28 polymorphism. It is important to keep in mind that atherosclerosis is a multifactorial disease that initiates early in life, involving the interplay of genetic and environmental factors. The lifestyle improvement is conditioned by environmental factors (such as nutritional behaviour and practice of physical activity) and may be particularly worthy in obese individuals with a less favourable genetic background.

Despite the new data reported here, this work presented some limitations. Obesity was defined according to CDC although a novel criteria is now recommended for the Portuguese population, causing us to have probably underestimated the degree of obesity. Nevertheless, at the beginning of this study the criteria recommend by the Portuguese Ministry of Health was that of CDC. Also, the number of controls was small to evaluate *UGT1A1* genotype distribution in cases and controls; actually, besides ethics requirements, parental approval in healthy children and adolescents is difficult to obtain. The evaluation of body composition by DEXA was also performed only in a subgroup of participants due to logistical constraints and equipment availability. Furthermore, we did not evaluate the association between bilirubin and the MS as a large proportion of our obese patients were under the age of 10, not allowing their classification according to the International Diabetes Federation (IDF) definition.

In conclusion, body fat percentage is a major determinant of TB levels independently of UGT1A1*28 polymorphism in obese children and adolescents. This may have a particular relevance, as obese individuals, particularly those with 6/6 *UGT1A1* genotype and higher body fat mass, may benefit from a closer clinical follow-up, considering their increased risk for other comorbidities. Moreover, lifestyle modifications at low ages, when good habits can be created, should be highly encouraged in such obese children and adolescents.
